# Coiled Plant Tendril Bioinspired Fabrication of Helical Porous Microfibers for Crude Oil Cleanup

**DOI:** 10.1002/gch2.201600021

**Published:** 2017-03-20

**Authors:** Yueyue Zhao, Xiaran Miao, Jinyou Lin, Xiuhong Li, Fenggang Bian, Jie Wang, Xiangzhi Zhang, Baohua Yue

**Affiliations:** ^1^ Shanghai Institute of Applied Physics Chinese Academy of Sciences Shanghai 201203 China; ^2^ Department of Chemistry College of Sciences Shanghai University Shanghai 200444 China

**Keywords:** coaxial electrospinning, coiled tendril, helical porous fiber, oil sorption **·**

## Abstract

Fabrication of the helical fibers with sheath/core structure comprising 3D interconnected porous polystyrene (PS) and ductile polyvinylidene fluoride is inspired by coiled plant tendril. The key innovation point applied in this study is to produce a helical porous system based on sheath/core structure that can come into being a huge storage space in the sorption process for crude oil. More importantly, the mechanical properties confirm to have a more excellent improvement than that of the initial PS fibers, which make it become a possible candidate for the large‐area sorption and reuse of crude oil from the ocean or industry. The bioinspired fabricating strategy opens a significant avenue between the coaxial electrospinning and crude oil contamination cleanup. It is also expected that the unique structure can make it a promising candidate for applications in energy conversion, tissue engineering, and intelligent devices.

Crude oil, a valuable natural resource, has been a fundamental part of human life since the dawn of industrial civilization because it can greatly change people's life‐style applied in various fields including traffic, manufacture, warfare, and academic research. However, frequent oil leakage accidents in ocean have happened during the exploration and transportation processes of crude oil, usually bringing catastrophic effects to the marine ecosystem, and giving rise to serious waste of the nonrenewable resource.^1^, ^2^, ^3^ A high‐profile example is the 2010 Gulf of Mexico oil leakage, which released an estimated 4.9 million barrels of crude oil into the ocean, causing a colossal disaster for marine animals and organisms. The long‐term catastrophe of marine ecosystem and the loss of resource by crude oil leakage call for an urgent need to develop advanced materials and technologies for the crude oil cleanup.

More recently, the most commonly used countermeasures for crude oil leakage remediation can be categorized into three main types including chemical methods (controlled burning, chemical dispersants, and the use of solidifiers),^4^, ^5^, ^6^ bioremediation,^7^ and physical methods (booms, skimmers, and sorbents).^8^, ^9^, ^10^ However, most of these methods only focus on cleaning up, ignoring the squander of crude oil resource and the introduction of other types of contamination during the remediation process. As an effective solution scheme, the use of sorbent materials has become one of the most attractive directions because it is convenient and not brings adverse effect to the environment. An excellent sorbent material should have hydrophobic and oleophilic properties, high oil sorption efficiency, long‐playing storage capacity (providing more time for transfer of crude oil), facile fabrication process, and low cost. However, traditional oil sorbents, including man‐made fibrous materials,^11^, ^12^ porous carbon‐based materials,^13^, ^14^, ^15^ and synthetic materials, experience various problems like low oil/water separation efficiency, poor oil storage capacity (sorption capacities: 2–30 times, draining time: 10–120 s), complicated preparation process, and high materials cost.^16^, ^17^, ^18^, ^19^ Therefore, it is imperative to search for a simple method to fabricate a type of span‐new material that could be used for cleaning up crude oil leakage, typically with inexpensive cost, facile fabrication process, and excellent oil cleanup properties.

In the past decade, bioinspiration, served as an innovative source, could provide a wide range of ideas for the material scientists to fabricate various advanced materials with remarkable features such as ultralight porosity, hierarchical structure, and various biomimetic surfaces with superhydrophobicity and oleophilicity.^20^, ^21^, ^22^ Recently, Gerbode et al. reported such an interesting phenomenon with respect to how the cucumber tendril coils and overwinds.^23^ They revealed that some cirrus plants can wend its way to sunlight or their niches relying on surrounding obstacle. During climbing, these straight tendrils first find and attach to supports, and then the helical coiling would be formed and extended by repeated enwinding. More importantly, by experiment shown in **Figure** **1**a–c, we also unexpectedly found that there were numerous micropores in the interior of withered loofah vine tendril, compared to the young tendril. Based on these discoveries, the combination both the helical porous microfibers and the hydrophobic and oleophilic properties will be a significant solution for crude oil cleanup because comparing to the no‐helical fibers, the biomimetic helical fibers has not only the intrafiber pores, but also the interfiber cavities formed by the own helical coiling of fibers, which provides an enormous and efficient storage space for crude oil sorption. In this work, two types of appropriate polymers are proposed to prepare the biomimetic helical fibers. The porous polystyrene (PS) was added to form sheath of the physical model. The polymer polyvinylidene fluoride (PVDF) with a good ductility was utilized to be core of the model. As shown in Figure 1d–f, the as‐designed fibers exhibit a helical coiling shape. Meanwhile, it can be easily seen that these helical fibers present a huge cavity, as well as the coaxial and porous structure. In addition, the preparation method that coaxial electrospinning shown in Figure 1g, is also a simple process, without the need for complex and expensive equipment.

**Figure 1:**
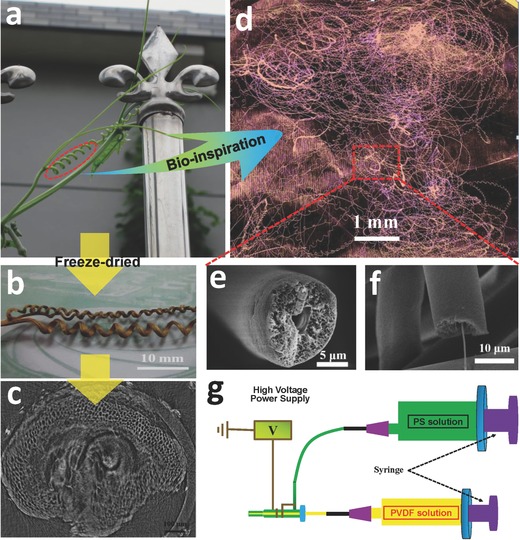
a) The parasitic coiled tendril of young loofah vine. b) The optical image of withered loofah tendril after freeze‐dried. c) Cross‐sectional CT image of freeze‐dried loofah tendril. d) The optical image of biomimetic plant tendril. e,f) Cross‐sectional and flank‐fractured scanning electron microscopy (SEM) images of the helical porous fibers. g) Schematic showing the fabrication method of helical porous fibers.

Some previous literatures have reported the related works with regard to the fabrication of helical fibers.^24^, ^25^, ^26^ However, it is difficult to clarify the formation mechanism. On the one hand, the fibers featured with helical coiling shape cannot steadily produce. On the other hand, the formation of helical coiling is a very elusive process. Herein, based on the existence of vast helical fibers, the prepared process of helical fibers with sheath/core structure is exhibited. As shown in **Figure** **2**, in general, there are four different stages. First, the polymer drop held by its surface tension at end of nozzle. Meantime, charge is induced on the liquid surface by the electric field, which causes a force directly opposite to the surface tension. As increasing the intensity of the electric field, the drop is elongated to form a conical shape known as the Taylor cone.^27^ When the repulsive electrical force overcomes the surface tension force, a charged jet is ejected from the tip of Taylor cone. Second, the charged jet travels in air, and its trajectory can be controlled by coaxial electric field. The electric field distribution makes the charged jet present periodic swing (Figure 2a). Third, as time goes on, the solvent rapidly evaporates (insets of Figure 2a), and the charge is also gradually disappearing. The outer sheath (PS) starts to come into being a 3D interconnected porous structure. Then the forming fiber is continuously traveling, and the inner elongated core with excellent scalability occurs to shrink, which induces the formation of helical shape (Figure 2b). Finally, the helical fibers are collected by different collecting devices, respectively. It can keep the primary shape when the helical fibers are deposited on the slab (Figure 2b), whereas the helical coiling are straightened when collected by the rolling drum (Figure 2c).

**Figure 2:**
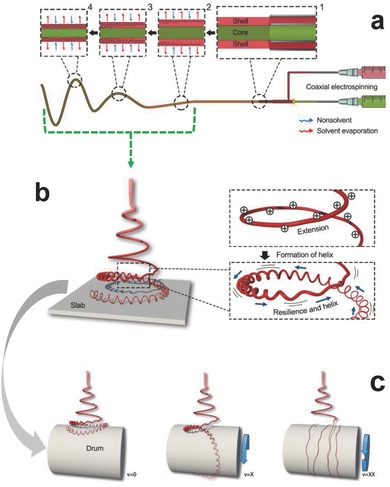
a–c) Schematic showing the formation mechanism of helical porous fibers.

The helical porous fibers with sheath/core structure presented in the process is induced by various factors like the properties of materials, collecting device, and electric field distribution, as well as the conductivity of solutions discussed in previous literatures. First, a different shrinkage behavior of PVDF and PS can contribute significantly to the formation of helical shape in bicomponent fibers. When the charged jet is elongated in the electric field, two types of polymers undergo a series of instabilities and solidification, and the straight fiber gradually transform into a helical coiling shape. The reasonable interpretation is that a right balance has to exist between the longitudinal compressive force and the rigid resistance for helical formation, which are arisen from the ductile PVDF and the stiff PS, respectively. Second, the substrate also plays an important role in the collection process of helical fibers. From Figure 2b,c, there are two different collection devices. The fibers deposited on a complanate substrate can keep the helical shape, whereas the straight fibers come into being when substituted by the rolling drum. The transformation of helical fiber into straight shape can be mainly attributed to the fact that the revolving tension derived from the rotation of drum exceed the twisty force of helical fibers when they contacted with substrate each other, which can agree well with the previous reports.^28^ In addition to the above, the electric field, as a basis for coming into being the continued fibers, provided a stable spinning process. Because of the entanglement of chains in the charged fluid, the fluid does not break up into droplets but forms a stable jet when the electrostatic repulsive force on the fluid surface overcomes the surface tension. More importantly, the electric field induced by coaxial needle creates a symmetrical distribution, which probable play a vital role in the formation of continuous helical fibers by conducting periodic motion.

**Figure** **3**a–f displayed several cross‐sectional SEM images of the pure PS fiber and the helical porous fibers featured with different cavities. For the helical porous fibers with sheath/core structure, the results manifested that the size of cavities could be adjusted by the solutions feed rate ratio of outer and inner pipelines based on the experiment. It can be easily seen that the diameter of cavities decreased as increasing feed rate of the outer pipeline. The size of cavities can be approximately equal to that of core fibers when the solutions feed rate ratio of outer and inner pipelines achieved 7:7 shown in Figure 3d and the inset of Figure 3d. To better understand the experimental finding, we attempted to make a theoretical calculation with respect to the size of cavities in the composite system via mathematical modeling on the basis of this fundamental experimental and theoretical analysis. The modeling is a straightforward congruent relationship between the size of nozzle, the fibrous diameter, and the size of cavity, typically expressed as

(1) $$\frac{S_{e}}{S_{c}}=\frac{D_{e}^{2}}{D_{c}^{2}-d_{c}^{2}}$$

where *S*_e_ and *S*_c_ are the sectional areas of nozzle and sheath of coaxial nozzle, respectively; *D*_e_ is the diameter of PS fiber, *D*_c_, *d*_c_ are the external and inner diameters of the coaxial PS fiber, respectively. The equation gives a facile mathematical statement, which neatly summarizes the relationship between several variables. The detailed interpretation is elucidated in Figure S1 (Supporting Information) and its relative deducing process. Figure S2 (Supporting Information) gives the calculated and measured values of the cavities. From it, it can be obviously seen that the calculated values can agree well with the measured one.

**Figure 3:**
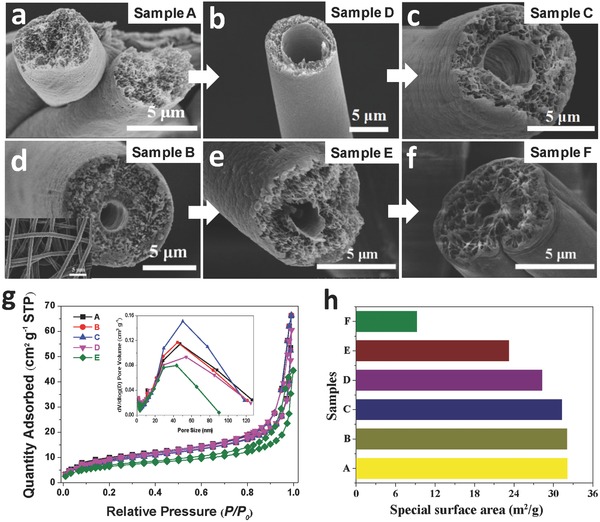
Cross‐sectional SEM images of a) the as‐prepared PS fibers, and composite fibers electrospun from the 6 wt% PVDF/DMF solution (core) and 30 wt% PS/DMF solution (sheath), the feed rate ratios of sheath/core are b) 1/7, c) 2/7, d) 7/7, respectively, the inset of Figure 3d shows the SEM image of PVDF fibers electrospun from the 6 wt% PVDF/DMF solution. e,f) Cross‐sectional SEM images of the composite fibers electrospun from the 6 wt% PVDF/DMF solution and 30 wt% PS/(mixture of DMF/THF) solution with 3/1 (DMF/THF) and 2/2 (DMF/THF) weight ratios in 1/1 feed rate ratio of sheath/core. The pure PS fibers and these as‐prepared fibers from different fabrication conditions are orderly named A, D, C, B, E, and F, respectively. g) Nitrogen adsorption–desorption isotherms and pore size distribution curves of the as‐prepared fibers. h) The theoretical special surface area curve of various fibers (A–F) calculated from the nitrogen adsorption–desorption isotherms by using the BET method.

In order to better illustrate the special surface area and pore scale of the helical porous fibers. The nitrogen physisorption (adsorption–desorption) isotherms and pore size distribution curves are shown in Figure 3g and Figure S3 (Supporting Information). The result exhibited that the isotherms could be categorized as two types with a distinct hysteresis loop, indicating the existence of mesopores (2–50 nm) and macropores (>50 nm) within these bionic fibers defined by the International Union and Applied Chemistry. Hence, the sharply increased quantity of N_2_ adsorbed at a high relative pressure (>0.8 *P/P*_0_) mainly resulted from the macropores and mesopores only grown on the outer and inside of PS fibers because the PVDF fibers were nonporous in cored position. The insets of Figure 3h and Figure S3 (Supporting Information) clearly displayed that the pore diameter was in the range of 2–100 nm, which can agree well with the N_2_ physisorption isotherm data as well as the observation of SEM image (Figure 3a–f). In addition, it is worth noting that there is a significant difference between curve of the sample F and that of other helical porous fibers in the nitrogen physisorption (adsorption–desorption) isotherms and the pore distribution. It is likely to be the reason that the rapid solvent evaporation hinders the formation of smaller nanopores, as increasing the THF component of solution. Figure 3h exhibited that the variation tendency of the special surface areas of as‐designed various fibers and pure PS fibers. The result demonstrated that the special surface area continued to decrease from the sample A to sample F, which was mainly caused by the size of fibrous cavity and porous distribution. The special surface area of the helical fibers sharply decreased when the weight ratios of *N,N*‐Dimethylformamide (DMF)/tetrahydrofuran (THF) mixtures were approximated to 2/2, which suggested that massive nanopores disappeared under the influence of bicomponent solution comprising DMF and THF.

On the other hand, as shown in **Figure** **4**, the surface morphologies of the as‐designed helical fibers with sheath/core structure were also presented. From Figure 4a, there are numerous nanopores on the surface of the helical fibers. It can be evidently seen that the pores with an average diameter of ≈20 nm packed on the fibrous surfaces as a result of the rapid phase separation induced by solvent evaporation and subsequent solidification.^29^ The pores on fibrous surfaces disappeared (Figure 4b) as increasing THF, but wrinkled surfaces emerged when the solvent mixture with 50% THF was used (Figure 4c). Generally, the difference in the apparent morphology of these as‐prepared fibers can be attributed to the competition between the surrounding moisture and the phase separation resulting from the mutual diffusion of the bicomponent solvents in electrospun process.^30^ The two types of surface morphologies obtained by adjusting the weight ratios of DMF/THF are similar to the that of real plant tendrils in nature shown in Figure 4d,e. In addition to the above two types of morphologies of coiled plant tendrils, among climbing plants also have the coiled plant tendril featured with hairiness, the unusual morphology species is likely to have a high‐efficiency isolation in restraining the atmospheric particle pollution. As shown in Figure S4 (Supporting Information), the biomimetic simulation also makes some progress, and the further breakthrough still continues. Of course, real tendril fiber ribbons are an elusive synthesis. Therefore, it is a long road in the fabrication of biomimetic plant tendril.

**Figure 4:**
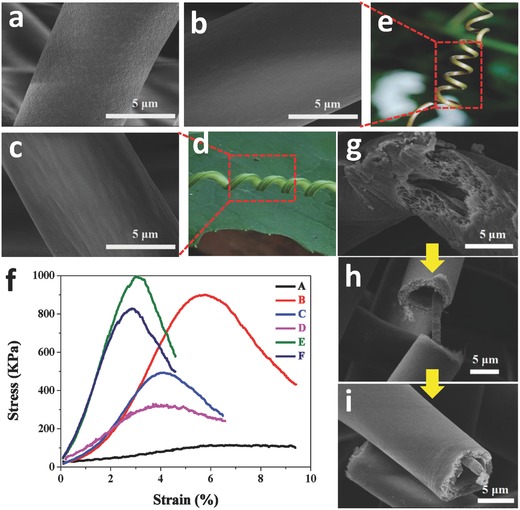
SEM images of the helical porous fibers with high magnifications in feed rate ratio of sheath/core: 1/1, electrospun from the 6 wt% PVDF/DMF solution and 30 wt% PS/(DMF/THF) solution with weight ratios of DMF/THF: a) 4/0, b) 3/1, c) 2/2. d,e) The morphology images of two types of real plant tendrils in nature. f) The typical stress–strain curves for the as‐prepared fibers. g–i) Cross‐sectional SEM images of helical porous fibers during the fracture process of the stress–strain test.

We also investigated the mechanical properties of these helical porous fibers with sheath/core structure via the stress–strain measurement for these samples. As shown in Figure 4f, it is easily seen that the mechanical strength of these helical porous fibers showed a more excellent improvement than pure PS fiber. Surprisingly, compared to other two types of helical porous fibers (samples E and F), the ductility of this helical fiber featured with numerous surface pores (sample B) partly enhanced under the stress–strain measurement. The phenomenon that the simultaneous increase of strength and ductility is abnormal but highly desired for applications because the mechanical strength and ductility are mutually exclusive in most materials.^31^ One possible reason is that the deformation of surface pores results in the increase of stretch. Of course, to better understand fractured process, we clearly exhibited the cross sections of helical porous fibers with sheath/core structure during the stress–strain test. As shown in Figure 4g–i, there are three main tensile stages in the fractured process. The first stage is the transformation of the helical fiber into a straight fiber, and the deformation occurred rapidly under slight tension. Afterward, the porous PS fibers were snapped with increasing tensile. Last, the inner PVDF fibers with high mechanical strength ceaselessly tightened until pulled off under higher tension.

The 3D helical shape, interconnected porous structure, and hydrophobic and oleophilic properties make the as‐designed helical fibers an excellent candidate for crude oil sorption. Based on our previous oil sorption measurement,^32^ herein, crude oil was selected as an adsorbed oil contamination. As illustrate in **Figure** **5**a, when the helical porous fibers were brought into contacting with dynamic oil/water system, It can absorb the crude oil easily, and the nearly 100% separation efficiency was achieved. More importantly, from Figure 5b, it can be obviously seen that the adsorbed oil was kept for a long time, which suggested that the oil had plenty of time to be transferred and reused. The result can be mainly attributed to the helical porous structure of fibers, which plays a vital role in locking oil. To investigate the maximum crude oil sorption capacity of the helical porous fibers, the sorption test was performed on the pure oil medium. Figure 5c shows that the helical porous fibers with sheath/core structure have a high sorption capacity for crude oil, compared to the corresponding straight fibers. Meanwhile, the mechanical equilibrium of oil sorption process is presented by a simulated diagram shown in Figure 5d. The coiling‐like structure plays a vital role in preventing oil draining. Among these helical fibers featured with sheath/core structure, it is easy to see that the sample D exhibits the best adsorbing capacity for crude oil. This is mainly attributed to its larger inner cavity. In general, these as‐designed fibers with coiling‐like structure can uptake crude oil at 37 to 69 times its own weight relying on the size of inner cavity. The result showed the as‐designed helical fibers have much higher sorption capacity for crude oil than some previously reported sorbents,^9^ such as wool‐based nonwoven (9–15 times), carbon soot (30–50 times), most vegetable fibers (below 10 times), and polymers (5–25 times).^33^ Of course, the sorption capacity of the helical fibers is lower than that of graphene and CNT‐based aerogels (50–350 times).^19^ However, the production method for the helical porous fibers is simpler and its fabrication cost is also cheaper, compared to that of graphene and CNT‐based aerogels. As another excellent property, it is noted that the relatively outstanding mechanical strength can make the helical fibers support the larger gravity. It means that our helical fibers used as a type of sorbent are a cost‐effective and promising substitution for the facile cleanup of crude oil spillage and chemical leakage from the ocean or industry.

**Figure 5:**
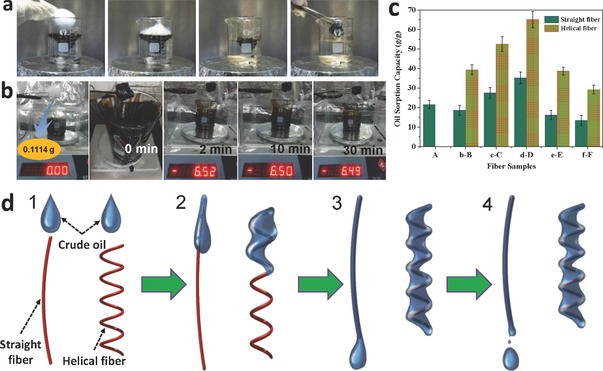
Sorption of crude oil by the helical porous fibers. a) Photographs showing the crude oil sorption process of the helical porous fibers from oil/water system. b) Photographs showing the crude oil desorption process of the helical porous fibers from pure oil system. c) Sorption efficiency of the various helical fibers (B, C, D, E, F) and their corresponding straight fibers (b, c, d, e, f) for crude oil. d) Schematic showing crude oil sorption mechanism of the helical fibers, compared to the straight fibers.

In summary, inspiring by coiled plant tendril, based on a fundamental experiment and theoretical analysis, we have demonstrated the first example in regard to the helical porous fibers with sheath/core structure utilizing two types of functional polymers comprising porous PS and ductile PVDF. Compared to those previous literatures only forming helical fibers or coaxial fibers, the breakthrough of biomimetic helical fibers is a span‐new creation. Several properties including special surface areas, architectural structures, surface morphologies, pore distributions, and mechanical property are systematically characterized by various instruments. The result manifested that these as‐designed helical fibers featured with higher special surface area, and uniform interconnected nanopore distribution. More importantly, their mechanical properties confirmed by the stress–strain measurement also made a more excellent improvement than that of the initial PS fibers. Meanwhile, the establishment of theoretical calculation provided a foundation for quantifying the helical porous fibers with different cavities. The helical porous fibers, used as an economic and efficient sorbent material of crude oil, are highly promising for environmental and ocean protection. The bioinspired fabricating strategy opens a significant avenue between electrospun fibers and oil contamination cleanup. We also expect that these unique properties can make it a great candidate for applications in energy conversion, tissue engineering, and intelligent devices in the near future. We also expect that the unique structure can make it a promising candidate for applications in energy conversion, tissue engineering, and intelligent devices.

## Supporting information

As a service to our authors and readers, this journal provides supporting information supplied by the authors. Such materials are peer reviewed and may be re‐organized for online delivery, but are not copy‐edited or typeset. Technical support issues arising from supporting information (other than missing files) should be addressed to the authors.

Supplementary
