# Supplementary material for: Coiled Plant Tendril Bioinspired Fabrication of Helical Porous Microfibers for Crude Oil Cleanup
Source: Glob Chall. 2017 Mar 20;1(3):1600021. doi: 10.1002/gch2.201600021 (PMC6607157; doi:10.1002/gch2.201600021)
Supplement: Supplementary file 1 — Supplementary [file GCH2-1-1600021-s001.pdf]

Copyright WILEY-VCH Verlag GmbH & Co. KGaA, 69469 Weinheim, Germany, 2017.

Global Challenges
Open Access

Supporting Information

for *Global Challenges,* DOI: 10.1002/gch2.201600021

Coiled Plant Tendril Bioinspired Fabrication of Helical Porous Microfibers for Crude Oil Cleanup

Yueyue Zhao, Xiaran Miao, Jinyou Lin, Xiuhong Li, Fenggang Bian,* Jie Wang, Xiangzhi Zhang,* and Baohua Yue*

Supporting information for *Global Challenges*

Coiled plant tendril bioinspired fabrication of helical porous microfibers for crude oil cleanup

Yueyue Zhao, Xiaran Miao, jinyou Lin, Xiuhong Li, Fenggang Bian,* Jie Wang, Xiangzhi Zhang* and Baohua Yue*

Shanghai Institute of Applied Physics, Chinese Academy of Science, Shanghai 201204, China.
E-mail: Jinyoulin82@gmail.com; Bianfenggang@sinap.ac.cn; Zhangxiangzhi@sinap.ac.cn

Part A: Experimental Section

Materials

Polystyrene (PS) (M_W = 350000 g mol^{-1}) was purchased from Aldrich. Polyvinylidene fluoride (PVDF) (M_W = 1000000 g mol^{-1}, Solef 6012) was purchased from America. N,N-Dimethylformamide (DMF) was purchased from Shanghai Chemical Reagents Co. Ltd., China. Tetrahydrofuran (THF) was purchased from Shanghai Chemical Reagents Co. Ltd. The crude oil was obtained from Daqing Oilfield China.

Preparation of electrospun solutions

PVDF used in this work was dissolved in DMF by 2 h to form 6 wt% precursor solution. Nearly 4 mL of precursor solution was placed in 10 mL syringe. In a similar way, Polystyrene solutions were prepared at concentration of 30 wt% by using mixture of solvents DMF and THF with various weight ratios of 4/0, 3/1, and 2/2, respectively. Then nearly 8 mL of precursor solution was placed in 20 mL syringe.

Coaxial electrospinning

The schematic of the coaxial electrospinning setup used in this work was shown in **Figure 1**g. The experimental set-up (ESM-CI, Shanghai YuYue Nano-technology Co. Ltd., China) is characterized by a coaxial nozzle, a high voltage power supply (Tianjin Dongwen High Voltage Power Supply Limited Company), two types of syringes (10 mL and 20mL), and two types of collecting devices (a roller and a slab). The coaxial nozzle consisted of the inner and outer chambers, which contained different polymer solutions. According to the features of coiled plant tendril from the

CT images (BL13W at Shanghai Synchrotron Radiation Facility) of freeze-dried loofa shown in **Figure 1**c, two types of polymers PS and PVDF were used as experimental materials. To study the forming mechanism of helical porous fibers featured with coaxial structure and accurately fabricate the biomimetic helical fibers, different collectors were equipped. We prepared six types of different fibers including a type of pure fibers electrospun from 30 wt% PS/DMF solution and five types of core/sheath fibers electrospun from the 6 wt% PVDF/DMF solution (core) and 30 wt% PS/DMF solution (sheath), the feed rate ratios of sheath/core are 1/7, 2/7, 7/7, respectively, as well as the core/sheath fibers electrospun from the 6 wt% PVDF/DMF solution and 30 wt% PS/(mixture of DMF/THF) solutions with 3/1 and 2/2 weight ratios in 1/1 feed rate ratio of sheath/core. The pure PS fibers and these helical porous fibers featured with coaxial structure from different fabrication conditions are orderly named A, D, C, B, E and F, respectively. During electrospinning process, the distance between the needle tip and collector kept 16-18 cm, and the voltage was set as 10 kV. All the experiments were carried out at 25 ^{o}C with the relative humidity of 40%.

Characterizations

The cross-sectional morphology of the coiled loofah vine tendril was observed by Computed Tomography (CT) (BL13W at Shanghai Synchrotron Radiation Facility). The morphology of the as-prepared electrospun fibrous mats was observed by a field emission scanning electron microscopy (FE-SEM) (S-4800, Hitachi Ltd., Japan); the Brunauer-Emmett-Teller (BET) surface area, pore volume, and pore width of the fibrous mats were characterized by nitrogen adsorption-desorption

measurements performed on an ASAP2020-HD88 analyzer (Micromeritics Co. Ltd.) at 25 ^{o}C. The tensile measurements were carried out on a Testing Machine (QJ-210, shanghai Qingji Instrument technology Co. Ltd) at room temperature.

Oil/water separation setup

The dynamic oil/water mixture (about 1/3, v/v) was formed by using a magnetic stirring apparatus shown in **Figure 5**a, apparatus, because the low density of oil and the high density of water easily separated the layers under quiescent conditions. The operating rotation speed imposed on the membrane was estimated to be about 100 rpm.

Crude oil sorption measurements

Approximately 0.12 g of sorbents were added into the glass beaker filled with 100 mL of pure crude oil placed on the scale (max: 1000.00 g). After 1h of sorption, the wet sorbents were drained for 30min. Crude oil sorption capacities for these sorbents were obtained from the following equation:

$$Q=\frac{m_s-m_0}{m_0}$$

where Q is the oil sorption capacity (g g^{-1}), m_s is the total mass of wet sorbent (g), m_0 is the mass of the sorbent before sorption (g). Each sample was measured three times, and the average value and standard deviation were calculated.

Part B. Supplementary figures, captions and discussions

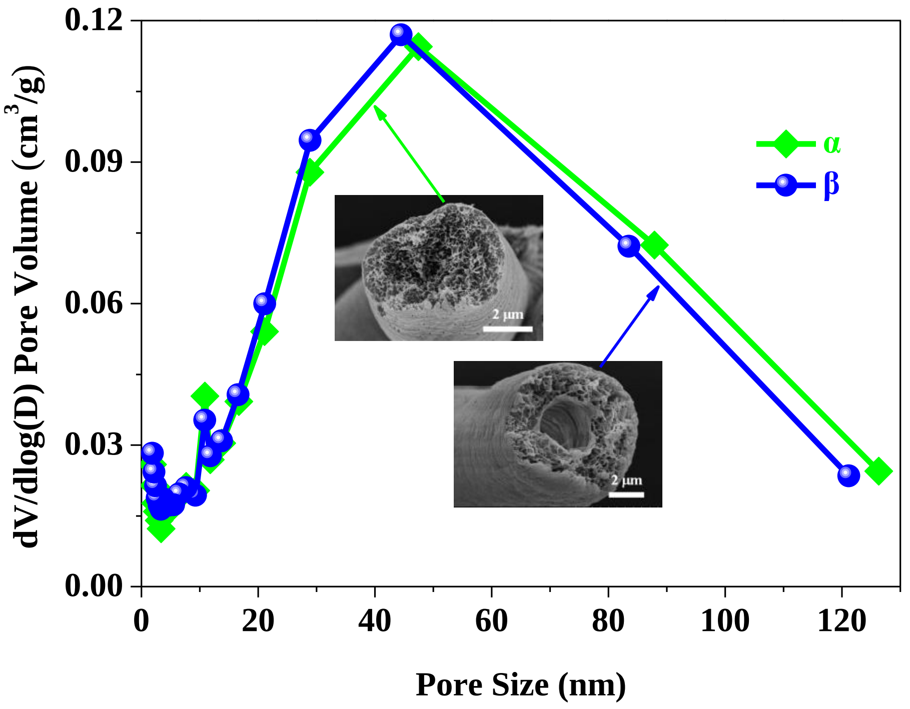

Figure S1. Pore size distribution curves of PS fibers calculated from the desorption branch using BJH method via different electrospun process in same feed rate: α) ordinary electrospinning, β) coaxial electrospinning.

As shown in **Figure** S1, for PS, it is worth noting that the intra-fiber pore distribution can be determined by the rate of jet comprising the mechanical rate and electric-field rate caused by the impetus and electric-field force, respectively. In other words, the fibrous density can be availably controlled by adjusting the jetting rate in the fabricating process. The derivation process as described below:

$$\boldsymbol{\rho_e = \rho_c} \tag{1}$$

$$\frac{\mathbf{m_e}}{\mathbf{v_e}} = \frac{\mathbf{m_c}}{\mathbf{v_c}} \rightarrow \frac{\mathbf{m_e}}{\mathbf{m_c}} = \frac{\mathbf{v_e}}{\mathbf{v_c}} \tag{2}$$

$$\mathbf{m_e = v_e \times t_{e\times} s_e \times c} \tag{3}$$

$$\mathbf{m_c = v_c \times t_{c\times} s_c \times c} \qquad (4)$$

$$\mathbf{(v_e = v_c,\ t_e = t_c)}$$

$$\mathbf{\frac{m_e}{m_c} = \frac{s_e}{s_c}} \qquad (5)$$

$$\mathbf{v_e = \pi \times \left(\frac{D_e}{2}\right)^2 \times L} \qquad (6)$$

$$\mathbf{v_c = \pi \times \left[\left(\frac{D_c}{2}\right)^2 - \left(\frac{d_c}{2}\right)^2\right] \times L} \qquad (7)$$

$$\mathbf{\frac{v_e}{v_c} = \frac{{D_e}^2}{{D_c}^2 \text{-} {d_c}^2}} \qquad (8)$$

$$\mathbf{\frac{s_e}{s_c} = \frac{{D_e}^2}{{D_c}^2 \text{-} {d_c}^2}} \qquad (9)$$

Where $\boldsymbol{\rho_e}$, $\boldsymbol{\rho_c}$; $\mathbf{m_e}$, $\mathbf{m_c}$; $\mathbf{v_e}$, $\mathbf{v_c}$; $\mathbf{t_e}$, $\mathbf{t_c}$, and $\mathbf{v_e}$, $\mathbf{v_c}$, are the densities, weights, electrospun speeds, electrospun times and volums of pure PS fiber and coaxial PS fiber, respectively; L is the total length of fibers; c is the concetration of PS solution; $\mathbf{S_e}$, $\mathbf{S_c}$ are the sectional areas of nozzle and sheath of coaxial nozzle, respectively; $\mathbf{D_e}$ is the diameter of pure PS fiber; $\mathbf{D_c}$, $\mathbf{d_c}$ are the external and inner diameters of the coaxial PS fiber, respectively.

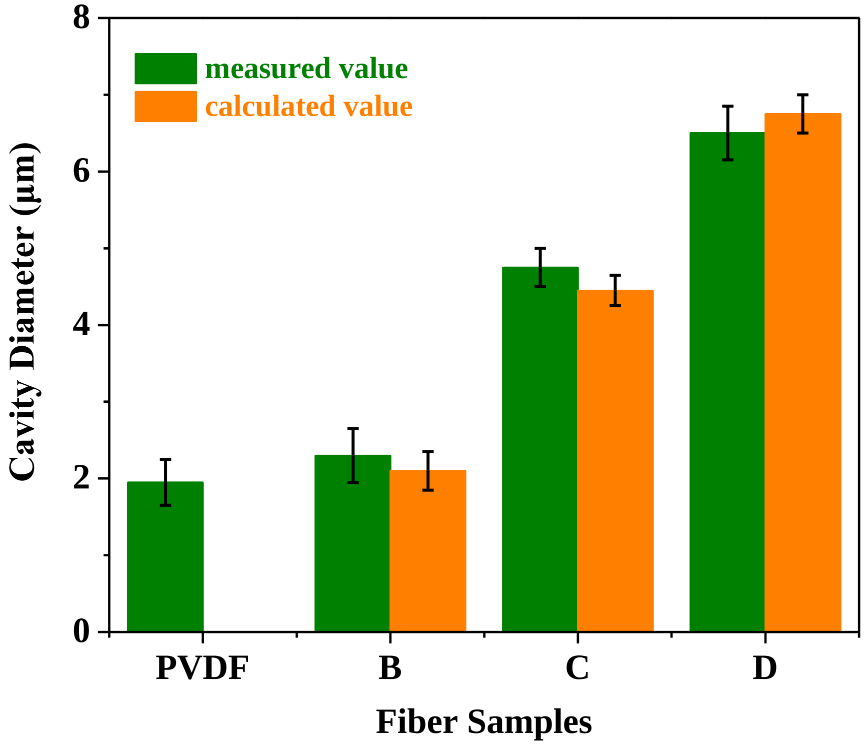

Figure S2. The diameters of various inner cavities from the as-prepared fibers in different feed rate ratios of sheath/core.

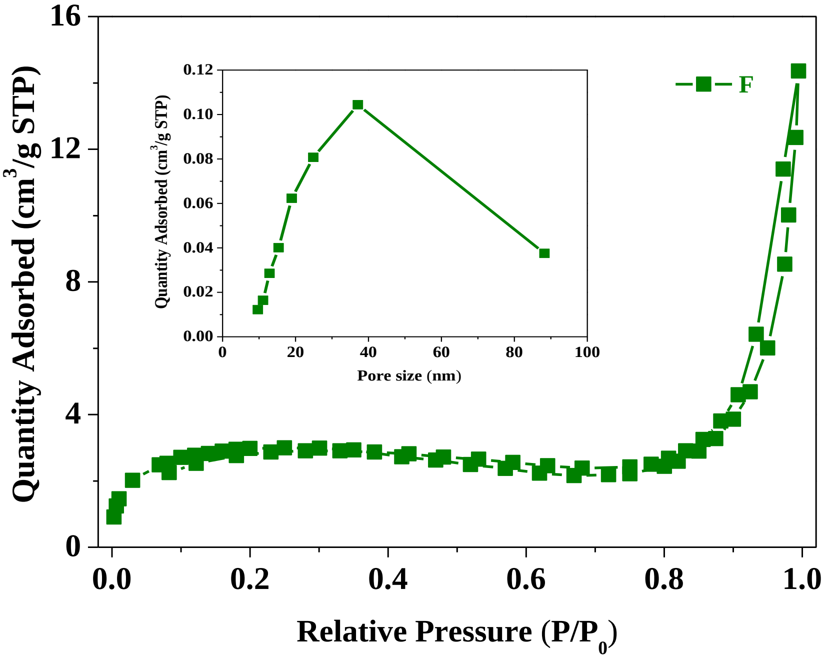

Figure S3. Nitrogen adsorption-desorption isotherms and pore size distribution curves of the helical porous fibers electrospun from 6 wt% PVDF solution in DMF and 30 wt% PS solution with weight ratio of DMF/THF: 2/2.

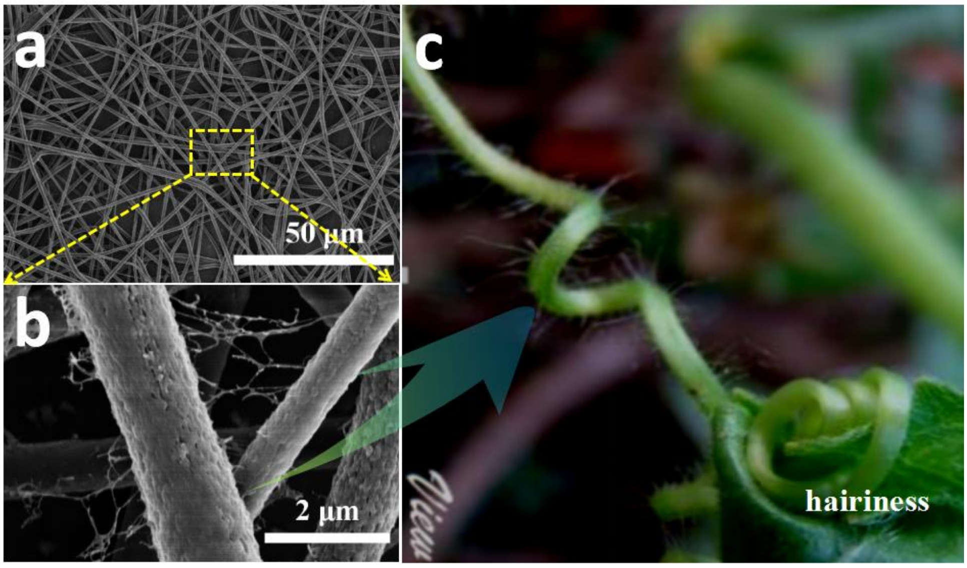

Figure S4. a, b) SEM images of PVDF fibers with different magnifications via electrospinning in special condition. c) The morphology image of real plant tendrils featured with hairness in nature.
